# Chronobiology of Melatonin beyond the Feedback to the Suprachiasmatic Nucleus—Consequences to Melatonin Dysfunction

**DOI:** 10.3390/ijms14035817

**Published:** 2013-03-12

**Authors:** Rüdiger Hardeland

**Affiliations:** Johann Friedrich Blumenbach Institute of Zoology and Anthropology, University of Göttingen, Berliner Str. 28, Göttingen D-37073, Germany; E-Mail: rhardel@gwdg.de; Tel.: +49-551-395414

**Keywords:** age-related diseases, aging, circadian, melatonergic agonists, melatonin, MT_1,_ MT_2,_ peripheral oscillators, polymorphisms

## Abstract

The mammalian circadian system is composed of numerous oscillators, which gradually differ with regard to their dependence on the pacemaker, the suprachiasmatic nucleus (SCN). Actions of melatonin on extra-SCN oscillators represent an emerging field. Melatonin receptors are widely expressed in numerous peripheral and central nervous tissues. Therefore, the circadian rhythm of circulating, pineal-derived melatonin can have profound consequences for the temporal organization of almost all organs, without necessarily involving the melatonin feedback to the suprachiasmatic nucleus. Experiments with melatonin-deficient mouse strains, pinealectomized animals and melatonin receptor knockouts, as well as phase-shifting experiments with explants, reveal a chronobiological role of melatonin in various tissues. In addition to directly steering melatonin-regulated gene expression, the pineal hormone is required for the rhythmic expression of circadian oscillator genes in peripheral organs and to enhance the coupling of parallel oscillators within the same tissue. It exerts additional effects by modulating the secretion of other hormones. The importance of melatonin for numerous organs is underlined by the association of various diseases with gene polymorphisms concerning melatonin receptors and the melatonin biosynthetic pathway. The possibilities and limits of melatonergic treatment are discussed with regard to reductions of melatonin during aging and in various diseases.

## 1. Introduction

Melatonin is primarily known as the hormone of the pineal gland, although its synthesis in additional extrapineal tissues and cells is documented by a large body of evidence [1]. In mammals, extrapineal melatonin is either poorly released to the circulation or only for short periods of time. Therefore, the circulating hormone mainly reflects its secretion by the pineal. Its chronobiological effects are, in mammals, mainly exerted by the pineal-derived fraction. Moreover, melatonin is not only released to the blood, but also, via the pineal recess, in relatively high initial concentrations, to the CSF [2,3]. Whether or to what extent these amounts secreted to the CSF contribute to circadian regulation is widely unknown.

In relation to the circadian system, melatonin is both an output and an input factor [4]. In brief, the mammalian pineal gland is controlled by the circadian pacemaker, the pair of suprachiasmatic nuclei (SCN), which receive photic information from the retina, in particular, from the melanopsin-containing retinal ganglion cells. The SCN stimulates melatonin biosynthesis only during scotophase, whereas light is inhibitory to this process. The information “darkness” is transmitted to the pineal via a neuronal pathway, which includes the paraventricular nucleus, an intermediolateral cell column of the upper thoracic cord, and a preganglionic sympathetic connection to the superior cervical ganglion, whose postganglionic fibers innervate the pineal [5]. Melatonin formation is stimulated (i) by β_1_-adrenergic activation of adenylyl cyclase and, thereby, protein kinase A, and (ii) α_1B_-adrenergic activation of phospholipase Cβ that leads to rises in Ca^2+^, protein kinase C and CaM kinases. With regard to the contribution of enhanced gene expression of the biosynthetic enzymes and their post-translational regulation, the mechanisms differ between mammalian taxa. Moreover, these processes are modulated by several peptidergic and glutamatergic mechanisms (summarized in [6]).

The nocturnal stimulation of melatonin biosynthesis leads to a high-amplitude circadian rhythm in the concentration of the circulating hormone, which further transmits the information “darkness” to virtually all, at least numerous, tissues [7,8]. The rhythmic input of enhanced melatonin to the various organs and CNS areas is, according to current evidence, not identical in mechanistic terms and seems to concern different levels of action. In some cell types, melatonin presumably only up- or downregulates certain genes, *i.e.*, exerts direct effects without necessarily affecting circadian oscillators. However, in the SCN, it modulates the oscillations in the pacemaker. This may affect both the amplitude and the phase of the primary oscillations. Changes in phase are known under the term of “chronobiotic” effects. Like other synchronizing signals, phase shifting by melatonin occurs according to a phase response curve, which has also been studied in humans [9,10]. In this role, melatonin acts as a feedback signal that contributes to the adjustment of the pacemaker to the external cycle and, presumably, to the maintenance of high-amplitude oscillations.

Contrary to earlier concepts, cellular circadian oscillations are not restricted, in vertebrates, to a pacemaker. As will be outlined next, oscillators based on same or similar cellular machineries as in the SCN are acting in numerous tissues, including various CNS areas as well as peripheral organs. The dependence of these oscillations on SCN-derived signals varies considerably among oscillators and some of them seem to be relatively autonomous [11,12]. With regard to the presence of melatonin receptors in remarkably many cell types [1], the question arises as to whether and, if so, to what extent the oscillators outside the SCN are influenced by the pineal hormone.

## 2. The Circadian Multioscillator System

The multiplicity of circadian oscillators in a mammalian body is remarkable. This diversity comprises different levels [12]: (i) oscillators are found in numerous organs, tissues, tissue explants and isolated cells; (ii) within an organ or CNS area, subpopulations of cells may constitute several oscillatory subsets, which can differ in their phase response to various time cues, a phenomenon that has even been observed within the SCN [12–14]; (iii) within a single organ and, perhaps, even a cell, parallel oscillators can exist, which operate on the alternate use of homologs or paralogs of core oscillator proteins and their respective genes. For example, the simultaneous expression of PER1 and PER2, frequently also of PER3, as well as CRY1 and CRY2, is a normal experience in the majority of tissues. The core oscillator protein CLOCK is sometimes replaced by its paralog, NPAS2. The consequence, namely multiplicity of oscillatory and regulatory properties, is even surpassed by the fact that the core oscillators are operating within a host of other regulatory proteins that interact with them and are differently expressed in the various cell types [15–17]. Several of them act as both output and back-feeding input factors of the core oscillator, others transmit external and intracellular information, e.g., concerning food or cellular energy status. Moreover, core oscillator proteins are thought to directly interact with other transcription factors to form a variable number of functionally diverse multi-protein complexes [17].

With regard to the fact that circadian oscillators are primarily cellular machineries, all nucleate cells may, in principle, be assumed to be capable of generating circadian oscillations. However, evidence exists for a substantial diversity concerning their autonomy. While some peripheral oscillators have been shown to possess a high degree of autonomy and to be relatively independent of the SCN and poorly or moderately influenced by the light-dark cycle [11,18,19], others are less autonomous [12] and may be regarded as slave oscillators of the SCN. In various cases, it has been observed that peripheral oscillations fade out under constant experimental conditions [12], in contrast to the long-lasting self-sustained oscillations of the SCN.

A full record of oscillators outside the SCN, which have been convincingly demonstrated or likely exist in the central nervous system and in peripheral organs, would exceed the scope of this article. The focus will rather be laid on those oscillators which have been shown to be modulated by melatonin or seem to be influenced indirectly by other actions of the methoxyindole. It should also be noted that the evidence for peripheral oscillators is not generally based in every case on cycles of all core oscillator proteins or their respective mRNAs, but is sometimes limited to rhythms in a few of them, such as PER1, PER2 and/or BMAL1. Central nervous oscillators outside the SCN have been demonstrated in the retina [20–27] and seem to exist, e.g., in the arcuate nucleus [28,29], the lateral hypothalamic area [29], dorsomedial nucleus [28], striatum [30,31], hippocampus [32] and solitary tract caudal brainstem nucleus [33]. Among brain-associated non-neuronal tissues, oscillators in the pars tuberalis (PT) [34–40] and the main part of the anterior pituitary [41,42] have to be mentioned. The PT oscillator should be of particular importance in seasonal breeders, whereas its role in other mammals remains to be clarified. Peripheral oscillators have been detected in adrenal cortex [43–46], pancreatic islets [47–49], exocrine pancreas [48], liver [29,41,50–60], adipose tissue [29,58,61], kidney [50,57], heart [50,52,58,62–65], blood vessels [65], muscle [29], stomach [59], oviduct [66], osteoblasts [67,68] and fibroblasts [69,70].

## 3. Chronobiological Effects of Melatonin outside the SCN

**Figure 1:**
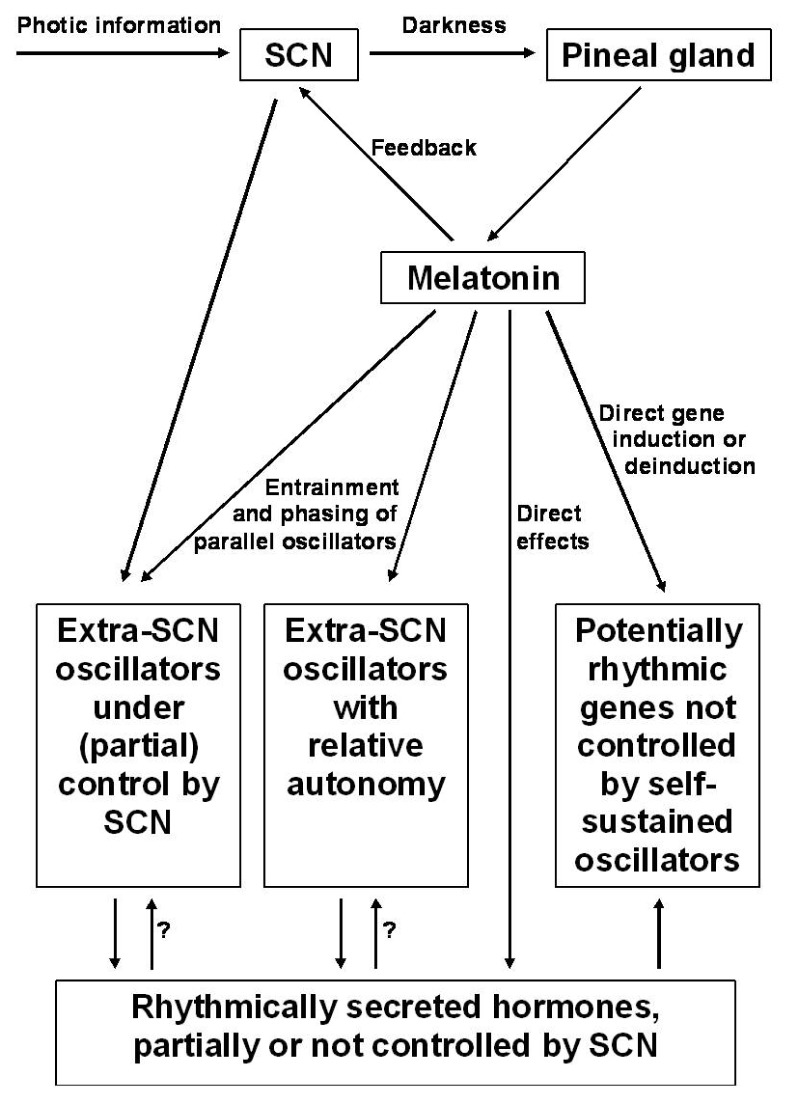
Schematic overview of the influences of melatonin on circadian oscillators of different degrees of independence from the SCN, additional effects by direct control of gene expression and the role of other hormones modulated by melatonin, such as glucocorticoids, insulin and some pituitary hormones.

Without detailed analysis, it is impossible to decide whether melatonin-induced changes in cellular parameters are based on the modulation of circadian oscillators and if so, at which regulatory level. In fact, a full spectrum of different possibilities seem to exist in a mammalian body, from direct effects not involving an oscillator to the modulation of oscillators with different degrees of independence from the SCN (Figure 1). Direct up- or downregulation of an enzyme does not *per se* require a cellular oscillator, although an overlap with additional effects mediated by a cellular clock cannot be generally excluded. Direct enzyme induction may be the case in glutathione peroxidase (GPx), which is upregulated by melatonin in numerous tissues, in both nocturnally active rodents and diurnally active birds [71,72]. In mammalian and avian brains, this enzyme peaks at night, but deviatant patterns have been described for other tissues [71]. However, GPx expression and activity are also influenced by other parameters, notably by the cellular redox balance, which can temporally differ between tissues. The direct involvement of melatonin is supported by strong reductions in GPx activity in pinealectomized rats [73] and the suppression of the GPx rhythm by light in chicken [74], findings that should not be expected if GPx activity would be primarily driven by a peripheral oscillator. Such a rhythm that is mainly produced by the periodicity of the inducer, melatonin, may secondarily generate other rhythms. This was assumed for the rhythm of glutathione reductase (GRd), which follows with a certain delay that of GPx, and is also abolished by light. GRd is known to be induced by a shift in the GSSG/GSH balance towards the oxidized form, which should occur as a consequence of *de novo* synthesized GPx. This is consistent with a GRd rhythm with a maximum appearing after that of GPx [72,74]. Whether or not these conclusions on direct or secondary inductions by melatonin or melatonin-mediated changes in the redox balance may also apply to the upregulation of several other antioxidant enzymes, remains to be clarified. This would especially concern γ-glutamylcysteine synthase, glucose-6-phosphate dehydrogenase, Cu,Zn- and Mn-superoxide dismutases and hemoperoxidase/catalase (reviewed in [1,75–77]).

Although the body of evidence for a role of melatonin in peripheral oscillators is still limited, several findings speak for such a conclusion. A case of particular importance is that of the oscillator in the adrenal cortex, because of the high-amplitude rhythm of glucocorticoid secretion generated there and the numerous effects of these hormones in other organs, in which they up- or downregulate countless genes. Enzyme induction and deinduction by glucocorticoids is classic biochemical knowledge, but it has been shown that these changes, especially in cyclically expressed genes, also depend in their extent on the circadian phase [78–80]. The importance of the glucocorticoid rhythm in the orchestrated phasing of peripheral oscillators has gained support by several recent studies. These hormones did not only favor the reentrainment of rhythms in various organs, in a tissue-specific manner [81], but also phase-shifted circadian gene expression in liver, kidney and heart, in the absence of corresponding effects in the SCN [82]. In cultured rat-1 fibroblasts, dexamethasone was shown to induce circadian oscillations in the expression of core oscillator and associated proteins [82]. Recently, using a luciferase reporter under control of the *Bmal1* promoter, circadian amplitudes were strongly enhanced and reset by co-culture with adrenal, but not thyroid or lung tissue [83]. Similar results were obtained by dexamethasone treatment of quiescent bone marrow mesenchymal stem cells [84]. On the other hand, glucocorticoids were also reported to interfere with food-induced phase-shifts of the peripheral oscillators in liver and kidney [85]. In the mouse lung, Clara cells were shown to possess a glucocorticoid-sensitive oscillator [86]. Collectively, these findings indicate that any alteration in the glucocorticoid rhythm, either caused by melatonin or resulting from its absence, would presumably lead to a plethora of secondary effects in the circadian multioscillator system.

Our understanding of the adrenal cortex oscillator has meanwhile considerably changed. The periodic synthesis and secretion of glucocorticoids is now known not to be just the consequence of the ACTH rhythm [44–46], and melatonin was additionally shown to interfere via the MT_1_ receptor with the ACTH-induced cortisol release [87]. Although this inhibitory action might indicate, at first glance, a general suppressive role of melatonin in the adrenal, the opposite is true, from a chronobiological point of view. This became evident in a comparison of the melatonin-proficient mouse strain, C3H, with the melatonin-deficient strain, C57BL [43]. In C3H, protein levels of PER1, CRY2 and BMAL1 were shown to oscillate with robust amplitudes, whereas C57BL exhibited only weak fluctuations and reduced expression of these proteins. These findings in the adrenal cortex strongly contrast with others obtained in the adrenal medulla, which is part of the sympathetic system and, thus, under direct neuronal control. In the medulla, the circadian clock was not substantially impaired by melatonin deficiency in C57BL [43].

However, neuronal control is not *per se* an indicator of melatonin independence. In fact, the murine retinal oscillator is strongly reminiscent of that one in the adrenal cortex, as far as the requirement of melatonin is concerned. Again, robust rhythms of PER1 and CRY2 levels were present in C3H mice, whereas C57BL mice did not show significant rhythms in these core oscillator proteins [22]. Comparisons between C3H and C57BL mice also revealed that the circadian rhythm of retinal dopamine damped out in DD in the melatonin-deficient strain, but not in C3H, a finding that also indicates a requirement of melatonin for the stable maintenance of peripheral cycling [88]. Daily injections of melatonin restored the retinal dopamine rhythm in C57BL. Similar findings were obtained for a rhythm in dopamine-dependent, PKA- and CAM kinase-mediated phosducin phosphorylation [89], which also damped out in C57BL but not C3H [26]. However, these results have also to be seen in the context of the intraretinal dopamine/melatonin antagonism [26,90], including the fact that they were obtained in rodents in which melatonin is synthesized in the retina [20,26], whereas this is not or only poorly the case in several other mammalian taxa, especially in primates including humans [91,92]. Therefore, it remains to be clarified whether eventually existing very low intraretinal melatonin levels or the circulating pineal-derived melatonin will be as important to a retinal oscillator in primates as in rodents.

Another neuronal oscillator outside the SCN, located in the striatum, was also shown to depend on the melatonin cycle. In mice, the circadian rhythms of *Per1* mRNA and PER1 protein levels were abrogated by pinealectomy [30]. In cultured murine wild-type striatal neurons, 1 nM melatonin downregulated *Clock* and *Per1* expression, and upregulated that of *NPAS2*, at the mRNA level, whereas *Bmal1* was weakly affected [31]. These changes were not observed in *MT_1_* receptor knockouts.

Another peripheral oscillator that is obviously influenced by melatonin is located in the pancreatic islet, which has also been in cultured explants [49,93]. Apart from increased levels of insulin secretion observed in *MT_1_* and *MT_2_* receptor knockouts as well as in respective double knockouts, the circadian rhythms of insulin transcripts as well as of circulating insulin were changed with regard to both phase and amplitude in the receptor-deficient animals [93]. Moreover, alterations of phase and amplitude were also described for the rhythms of *Per1*, *RevErbα* and *Dbp* expression in both pancreas and liver [93]. The influence of melatonin on other peripheral oscillators awaits further clarification. Studies on double knockouts may be regarded as a first-line approach to study this question.

The skin is another organ in which several cell types exhibit endogenous oscillations of clock gene expression. In the murine skin, such rhythms were detected in keratinocytes and hair follicle cells [94]. At least in mice, these cutaneous oscillators are, however, SCN-dependent and fade out after ablation of the central pacemaker. In humans, rhythmic clock gene expression has been convincingly demonstrated in hair follicle cells [95,96]. At the mRNA level, *Per2*, *Per3*, *Dbp*, *Nr1d1* and *Nr1d2* oscillated with robust amplitudes, whereas those of *Bmal1* and *Npas2* exhibited only weak fluctuations [95]. Whether and to what extent these endogenous rhythms are influenced by melatonin remains to be clarified. If they are, as in mice, generated by a slave oscillator of the SCN, melatonin would be primarily relevant via its effects on the pacemaker. The situation is complicated by the cutaneous synthesis of melatonin and hormones otherwise known from the hypophyseal-pituitary-adrenal axis as well as the expression of melatonin receptors in the skin, mainly MT_1_[97–99]. However, the influence of melatonin via cutaneous receptors was assumed to be less dependent on the circulating hormone, but rather on a continuous exposure to skin-derived melatonin [97].

Apart from the evidence obtained in melatonin-deficient genotypes, pinealectomized animals and receptor knockouts, as described, melatonin may have an additional effect on peripheral oscillators, although, in this case, its action is not independent of the SCN. This concerns the circadian rhythm of core body temperature, which is believed to be generated at the SCN, but which strongly depends, in humans and presumably other diurnally active mammals, on the feedback by melatonin, which is involved in the nocturnal decrease of this parameter [100–102]. This view is supported by the observation that, in elderly subjects, a deteriorated rhythm of body temperature can be normalized by melatonin [103]. Newly emerging evidence reveals the relevance of the core body temperature to peripheral oscillators. By contrast with the relative resistance of the SCN to entrainment by temperature, the peripheral oscillators are more strongly influenced [104]. Earlier results first demonstrated a support of rhythmic gene expression by low-amplitude temperature cycles in cultured peripheral cells [105], findings that were later extended to synchronization [106]. Actually, the normal physiological temperature cycle is believed to also entrain various autonomous or semiautonomous oscillators *in vivo*[104,107]. Thus, the hypothermic effect of melatonin may, in diurnally active mammals including humans, contribute to phasing and maintenance of amplitudes of peripheral oscillators.

## 4. Melatonin Dysfunction

Melatonin dysfunction can either result from gene variants or be acquired during life, in the course of aging or as the consequence of a disease. With regard to the remarkable pleiotropy of melatonin, such deviations should cause numerous secondary effects, including chronobiological alterations, and have the potential of pathophysiological relevance.

Gene variants that are statistically associated with pathologies either concern the biosynthetic pathway of melatonin formation or melatonergic signaling. However, in all these cases, it should be kept in mind that these associations only reflect risk factors, which may not necessarily lead to a disease in the absence of other influences, e.g., combination with other risk factors or an unfavorable lifestyle. Among gene variants concerning melatonin biosynthesis, polymorphisms of the arylalkylamine *N*-acetyltransferase (*Aanat*) gene were found to be associated with major depression [108]. Meanwhile, a considerable number of hydroxyindole *O*-methyltransferase (=acetylserotonin methyltransferase, ASMT) variants is known, which largely differ with regard to enzyme activity [109]. In various cases, this should lead to strongly reduced levels of circulating melatonin. Several variants, in particular those with low or disrupted activity, were associated with autism spectrum disorders [109–112], ADHD (attention-deficit and hyperactivity disorder) [109,113], recurrent depression [114], bipolar disorder [109,115] and intellectual disability [109]. Polymorphisms in the *MT_1_* receptor gene (*MTNR1A*) were also found to be associated with ADHD [113], with schizophrenia [116], coronary artery disease [117], polycystic ovary syndrome [118] and calcium nephrolithiasis [119]. Considerable attention has been paid to the polymorphism of the *MT_2_* receptor gene (*MTNR1B*). Variants are thought to be involved in polycystic ovary syndrome [120], rheumatoid arthritis [121], progressive subtypes of multiple sclerosis [122] and, perhaps, adolescent idiopathic scoliosis [123,124]. Importantly, this polymorphism was also associated with metabolic disorders, from elevated fasting glucose and cholesterol to diabetes type 2, as documented in numerous studies (summarized in [12,125]).

A gene polymorphism was also studied in the case a melatonin receptor-associated protein, GPR50. This has been identified as a mammalian ortholog of the non-mammalian Mel_1c_ receptor [126]. However, GPR50 is incapable of binding the pineal hormone. This orphan receptor heterodimerizes with MT_1_, thereby preventing G protein coupling [127]. Whether the association of GPR50 variants with, e.g., bipolar and seasonal affective disorders and elevated fasting triglycerides has to be explained by interference with melatonergic signaling remains to be unequivocally demonstrated, because GPR50 also interacts with other proteins not related to functions of melatonin (*cf.*[125]).

Melatonin dysfunction can be assumed to exist if the nocturnal values of the circulating hormone drop below a certain threshold that does no longer warrant sufficient receptor activation. With regard to the differences in affinity between MT_1_ and MT_2_ receptors (pK_i_-values of about 10.09 and 9.42, respectively, in wild-type human receptors [125,128]), reduced melatonin levels should lead first to functional losses in MT_2_ signaling. This may vary with regard to deviating affinities of mutant receptor variants.

A frequently observed phenomenon is the reduction of nocturnal melatonin in the course of aging [129–132]. The interindividual variability of these losses in melatonin secretion is, however, high. Whilst nighttime values are in some elderly subjects almost indistinguishable from daytime levels, others maintain a fairly well pronounced rhythm with only moderate reductions of the nocturnal peak. Correspondingly, age-related losses have been also demonstrated in melatonin concentrations of human pineals, cerebrospinal fluid, saliva, and in the amounts of the major urinary metabolite, 6-sulfatoxymelatonin (summarized in [125]). As long as the melatonin rhythm is still detectable in an aging individual, a phase-advance of the nocturnal peak is often observed [131], which may be in accordance with the typical age-related shortening of the spontaneous circadian period, which leads, under synchronized conditions, to a change in the phase angle. Several factors can contribute to the reduction of melatonin secretion. In principle, visual input to the SCN, integrity of the SCN and the neuronal connection to the pineal can be affected [131,133–135] as well as the integrity of the pineal itself, which may become, in some cases, progressively calcified [136,137]. As soon as the functioning of the SCN is impaired, the lack of a strong feedback signal by melatonin may further aggravate the functional losses. It should be also kept in mind that the reductions in circulating melatonin may also have considerable consequences for peripheral oscillators, too, and also for oscillator-independent up- and downregulations of genes in many places of the body. Even in these latter cases, this would lead to losses of timing and, thus, temporal coordination of physiological parameters as required for the well-functioning of an organism.

Reductions of melatonin secretion are also observed in remarkably many diseases and disorders. It seems important to discriminate between changes that occur as a consequence of a disease and others that may be causative or contributing factors to disease development and/or progression. This distinction cannot yet be made with certainty in various cases, especially, in those in which the consequences of mutations in receptor genes have not been clarified in terms of changes in affinity and coupling to signaling pathways. Judgments may be more easily made in cases in which the melatonin biosynthetic pathway is impaired by mutations in the *Aanat* and *Asmt* genes. This seems to be especially possible in the recently described variants of the ASMT (HIOMT) protein, as far as they are exhibit strongly reduced enzyme activities [109]. From this point of view, the association of such variants with autism spectrum disorders, ADHD, bipolar disorder, and cases of intellectual disability has gained a new quality of interpretation. However, it still remains unclear why some other variants that posses approximately normal enzyme activities are sometimes also associated with such disorders. The required clarification may be achieved by investigating differences in regulation, in protein-protein interactions and by determining enzyme stability or proteasomal degradation rates.

Reductions in melatonin largely appear to be the consequence rather than cause in cases of Alzheimer’s disease (AD) and other forms of senile dementia [125,131,134,135,138–143], although the lack may further aggravate the disease. Typically, levels of melatonin are by far more strongly decreased than in age-matched controls. Frequently, the melatonin rhythm is, from a certain point on, almost abolished. These changes are often explained by a progressing neurodegeneration in the SCN. Reductions of 6-sulfatoxymelatonin, which would imply corresponding decreases in melatonin, were also observed in cases of age-related macular degeneration [144], which may result from a reduced visual input to the SCN associated by a weakening of the aging oscillator. Reduced melatonin was also observed in cases of another neurodegenerative form of dementia, Pick’s disease [139]. Eventual changes in the SCN remain to be studied.

Apart from various other diseases with reduced melatonin, in which either the pineal gland is damaged, e.g., by a hamartoma or a craniopharyngioma, or in which severe changes in the hormonal system are present, such as in hypergonadotrophic hypogonadism (summarized in [125]), a number of conditions exist in which decreases of the pineal hormone are observed, although the causal relationship is not obvious at first glance. This observation includes some neurological disorders, notably a subpopulation of schizophrenics [145,146] and patients with primary obsessive-compulsive disorder [147]. Although major depression is not generally associated with reduced melatonin secretion, this was found in cases of comorbidity with multiple sclerosis [148]. However, subforms of major depression may require a reinvestigation with regard to observed polymorphism of the *Aaanat* gene, as mentioned above.

Meanwhile, a relatively large body of evidence has accumulated for reductions of melatonin under stressful and painful conditions, however, with considerable interindividual variation. These include several forms of cardiac diseases (coronary heart disease, myocardial infarction, cardiac syndrome X) [149–155], fibromyalgia [125,156], neuralgia [156], migraine [157,158], severe epilepsy [159,160], Menière’s disease [161], bulimia [156], critical illness [162–164], postoperative stress [165], acute intermittent porphyria, especially during seizures [166,167], and cases of cancer [168,169].

Decreases in melatonin have been also observed in diabetes type 2 [170,171]. This is of particular interest with regard to the repeatedly demonstrated association of the *MT_2_* (*MTNR1B*) polymorphism and the effects of melatonin on islet function and the islet oscillator [49,93]. Therefore, the relationship between melatonin signaling and diabetes type 2 may be more profound than previously believed.

## 5. Consequences of Impaired Melatonergic Signaling and Treatment Options

To draw adequate conclusions, it seems necessary to distinguish between different conditions under which melatonergic dysfunction is observed. A major discrimination has to be made between disorders with and without SCN destruction. As soon as the SCN is damaged by neurodegenerative processes, e.g., in AD, melatonin will not be able to appropriately control the phase of the pacemaker and all SCN-dependent processes can be expected to deteriorate. Of course, intermediate stages do exist, in which disease progression is not too much advanced, such that melatonin may be partially effective. However, it may be a misconception to believe that, in cases of a dysfunctional SCN, any treatment with melatonin or synthetic melatonergic agonists would be *per se* useless. This conclusion is based on our actual understanding of the body’s circadian organization as a multioscillator system, which only partially depends on the SCN and which is, in some of its oscillatory elements, susceptible to various different time cues, including nonphotic ones and the message conveyed by melatonin, too. Therefore, melatonin signaling beyond a dysfunctional SCN may be of particular importance to maintain, as far as possible, a coordinated time structure within the organism, by periodic inputs into extra-SCN oscillators and by timely inducing or deinducing gene expression in cells that do not contain melatonin-sensitive oscillators but melatonin-controlled genes.

Unfortunately, this aspect has not been systematically investigated in AD patients, especially not with regard to vegetative functions. The focus of AD studies has usually been put, apart from cognitive parameters, mainly on sleep and behavioral abnormalities, such as sundowning. With regard to its circadian components, sleep is largely dependent on the SCN, but not entirely, and includes a melatonin effect transmitted by thalamic melatonin receptors that favors the generation of sleep spindles, in a thalamo-cortical interplay [172,173]. Whilst melatonin was found not to be generally effective as a soporific agent in AD [174], positive effects were described concerning both sleep improvements and alleviation of behavioral symptoms in a number of patients [175–177].

Under the other condition of a functionally preserved SCN, a further discrimination seems to be necessary. On the one hand, melatonin receptor variants with a deviatant or impaired signal transduction may, in principle, lead to similar (patho-)physiological consequences as reduced melatonin secretion, whether caused by a defective biosynthetic pathway or acquired by aging or disease. However, the options for treatment can be expected to strongly differ. Reduced melatonin levels may be partially corrected by treatment with melatonin or synthetic melatonergic drugs, whereas no such improvement can be achieved in cases of receptor dysfunction. One might believe that mutual functional substitution of MT_1_ and MT_2_ receptors, which has, in fact, been observed, may diminish this problem, but these findings cannot be generalized to all tissues and cell types. Differences in signaling pathways of the two receptor subtypes do exist in a cell-specific way (*cf.*[178]). Although the MT_2_ receptor, which has lower affinity to its ligand than MT_1_, may replace to a certain degree a dysfunctional mutant MT_1_ receptor and though administration of melatonin may partially compensate for the lower affinity, a complete readjustment cannot be achieved, for reasons of differences in the distribution of the subtypes and cell type-specific signaling pathways [178], which exceed the classic inhibition of adenylyl cyclase. In the extreme, activation of the receptor subtypes leads to opposite effects, such as in vasomotor control, where MT_1_ signaling causes constriction, but MT_2_ activation dilation [179].

It may still be too early to translate these general considerations into an advice for treatment. In particular, the actual body of knowledge on human melatonin receptor gene polymorphisms may shed light on relationships to etiologies of diseases and disorders, but it does not yet provide sufficient information on the molecular and cell biological consequences. To what extent gene variants or other SNPs that may alter receptor gene expression really cause quantitative changes in expression levels, alterations in G protein coupling and other downstream effects remains to be studied on a broader scale. A human variant of MT_1_ with significantly reduced binding capacity (*B*_max_) has been described in an earlier study [180]. A more recent investigation has identified a number of MT_2_ variants with altered binding and signaling properties [181]. Four variants were incapable of binding melatonin, 11 others did not show G_i_ signaling, another one was devoid of ERK activation, and numerous rare loss-of-function variants were also identified. Studies like this one should stimulate researchers to further investigate the cell biological consequences of receptor polymorphisms more systematically with regard to the various diseases and disorders associated with melatonin signaling. The identification of changes in expression and signaling is particularly important with regard to the options of treatment. Variants defective in melatonin binding and/or G protein coupling will be resistant to melatonin therapy. Moreover, variants should not only be seen under the aspect of disrupted function, since the possibility of an undesired receptor overexpression may likewise lead to pathological consequences. This is reflected by the actual discussion concerning the enhanced expression of the MT_2_ risk variant, the G-allele of the *MTNR1B* gene, for diabetes type 2 [182]. In fact, carriers of a gain-of-function mutant should not be treated with melatonin. Whether antagonists might be helpful in such cases, as suggested, remains to be clarified, especially with regard to the multitude of side effects that can be expected. Reductions in melatonin levels that are observed in diabetes type 2, as mentioned above, may already contribute to weakening of MT_2_ signaling. However, it should be noted that loss-of-function mutants of the *MTNR1B* gene were also associated with diabetes type 2 [181].

Relative to the genetic changes in melatonergic signaling, the situation appears to be much clearer in cases in which melatonin levels are decreased because of aging or disease, in the presence of a relatively well-functioning SCN. Even if the focus of this article is mainly laid on the extra-SCN oscillators and peripheral effects not requiring an oscillator, the ensemble of clocks in the multioscillator system may be more easily orchestrated as long as the pacemaker is sufficiently working.

Regarding this condition, the major question is that of whether a replacement therapy can be achieved. A major obstacle at this aim is the short halflife of circulating melatonin, which is mostly in the range of 20–30 min, sometimes less, but maximally about 45 min [173,183]. Two different attempts have been made to circumvent this problem, the use of controlled-release tablets of melatonin and of synthetic melatonergic agonists with a longer halflife. Since the properties of the respective pharmaceutical products have been repeatedly reviewed on a comparative basis with regard to efficacy, pharmacology and metabolism [125,173,184–187], these details will only be briefly discussed. The indolic agonist β-methyl-6-chloromelatonin (TIK-301) [188] has a halflife in the range of 1 h and, compared to melatonin, an approximately same affinity to MT_1_ but a higher one to MT_2_. The naphthalenic agonist agomelatine has a halflife in the range of 1–2 h and affinities to both receptors slightly above those of melatonin [189]. Another nonindolic agonist, ramelteon, also exhibits a halflife of 1–2 h, but has higher affinities to both receptors [128,190]. However, one of the major metabolites of this latter compound retains some 10% of the receptor affinities, but has a halflife 2–5 h longer than the parent drug, properties that result in metabolite concentrations 20–100 times higher than ramelteon [181,191]. Properties of other, still investigational agonists have been summarized elsewhere [125,192,193].

To date, the value of these drugs cannot be judged with certainty based on nocturnal time profiles of these compounds and their metabolites nor of effects in peripheral tissues. The endpoints tested are almost exclusively related to the central nervous system and mainly concern sleep, but also the readjustment of circadian functions with focus on SCN-dependent effects, and to antidepressive actions that largely extend to subtype-specific serotonergic antagonisms. Sleep may be taken as a physiological function that might be suitable for judging on complete efficacy in a substitution therapy. The general observation is that all melatonergic drugs are effective in reducing sleep latency, but that all of them, despite some statistically demonstrable improvements, are not capable of fully restoring sleep maintenance [125,186]. This may be indicative of insufficient efficacy in terms of a substitution therapy.

Nevertheless, the actual inability of satisfactorily restituting a nocturnal melatonin profile that both avoids strongly supraphysiological levels after drug intake, with the eventual consequence of receptor desensitization [178], and an untimely drop of the circulating agonist in the second half of the night, should not retain researchers from investigating the effects of melatonin on peripheral functions in elderly or diseased individuals. For resychronizing the pacemaker, relatively small amounts of immediate-release melatonin or other melatonergic drugs are sufficient [125,186]. Therefore, a chance may exist to likewise resynchronize peripheral and other extra-SCN oscillators by short-acting melatonin pulses. This should be seen as a possible way of improving the functioning of a gradually deteriorated multioscillator system, even under conditions of a partially defective pacemaker. The feasibility of this concept remains to be studied.

## 6. Conclusions

Various lines of evidence have shown that melatonin plays a role in the appropriate functioning of several extra-SCN oscillators. This includes results from melatonin-deficient animals, receptor knockouts and phase-shifting experiments in explants. These investigations have not yet been extended to a larger number of oscillators. Nevertheless, systematic studies into this direction seem to be worth-while, promising and, in this author’s belief, necessary. As a major orchestrating chronobiological signal molecule, melatonin may be effective in coordinating numerous rhythms. Progress in this regard may open our view on the intraorganismal timing within the multioscillator system. Moreover, this kind of research has the potential for improving the persistence, amplitudes and coordinated phasing of oscillators under conditions of melatonergic dysfunction. This may be of particular value under conditions of reduced melatonin, as it occurs in elderlies and in various pathologies. Recent data on defective melatonin biosynthesis provide cases in which administration of melatonin or other melatonergic drugs seems recommendable. Moreover, melatonergic treatment may be helpful in patients in whom the SCN is poorly functional, because of neurodegeneration or impaired light input. It is the hope that this may help maintain a minimum of rhythmic time structures in individuals who lack a sufficiently strong circadian signal originating from the SCN.

At the present state of knowledge, a complete replacement therapy has not been achieved, if possible at all. A desiderate may be to reconstruct a normal nocturnal time profile of melatonin. Whether or to what extent this will be really necessary, as long as only chronobiological effects are intended, remains to be clarified. Synchronizing effects may already be attained at relatively moderate doses of immediate-release melatonin, as known from SCN-dependent rhythms. This is the more important as long-term tolerability will be a decisive issue for such treatments. The toxicological concerns related to the use of synthetic agonists have also to be taken into consideration [125,186,187]. There are additional physiological or pathophysiological limits to the use of melatonin and synthetic melatonergic agonists, as discussed, for example in the case of the G-allele of the *MTNR1B* gene, and various other conditions which have been identified or, at least, assumed to have unfavorable consequences [125,173,187]. In general, however, melatonin is mostly well tolerated, and low, chronobiologically effective doses should be preferred in comparison to the much higher recommended doses of the synthetic agonists.
